# Complex Commingling: Nucleoporins and the Spindle Assembly Checkpoint

**DOI:** 10.3390/cells4040706

**Published:** 2015-11-03

**Authors:** Ikram Mossaid, Birthe Fahrenkrog

**Affiliations:** Institute of Molecular Biology and Medicine, Université Libre de Bruxelles, Charleroi 6041, Belgium; E-Mail: Ikram.Mossaid@ulb.ac.be

**Keywords:** nuclear pore complex, nucleoporins, spindle assembly checkpoint, mitotic checkpoint, mitosis, kinetochore

## Abstract

The segregation of the chromosomes during mitosis is an important process, in which the replicated DNA content is properly allocated into two daughter cells. To ensure their genomic integrity, cells present an essential surveillance mechanism known as the spindle assembly checkpoint (SAC), which monitors the bipolar attachment of the mitotic spindle to chromosomes to prevent errors that would result in chromosome mis-segregation and aneuploidy. Multiple components of the nuclear pore complex (NPC), a gigantic protein complex that forms a channel through the nuclear envelope to allow nucleocytoplasmic exchange of macromolecules, were shown to be critical for faithful cell division and implicated in the regulation of different steps of the mitotic process, including kinetochore and spindle assembly as well as the SAC. In this review, we will describe current knowledge about the interconnection between the NPC and the SAC in an evolutional perspective, which primarily relies on the two mitotic checkpoint regulators, Mad1 and Mad2. We will further discuss the role of NPC constituents, the nucleoporins, in kinetochore and spindle assembly and the formation of the mitotic checkpoint complex during mitosis and interphase.

## 1. Introduction

Nuclear pore complexes (NPCs) are large protein complexes that are embedded in the nuclear envelope (NE), thereby spanning the inner nuclear membrane (INM) and the outer nuclear membrane (ONM) [1]. These giant structures constitute the sole site for macromolecular exchange between the cell nucleus and the cytoplasm in eukaryotic cells [1]. Among distinct species, NPCs vary in their molecular mass and in their number per nucleus: the molecular weight of NPCs in human cells and *Xenopus laevis* oocytes is about 110 MDa [2,3], while yeast NPCs have an estimated mass of ~66 MDa [4,5]. The number of NPCs is estimated to be about 4000 NPCs/nucleus in human cells [2], 5 × 10^7^ NPCs/nucleus in mature *Xenopus* oocyte nuclei [6], and about 200 NPCs/nucleus in yeast [4]. Despite these differences in molecular weight and number, NPCs from distinct species share a roughly tripartite structural organization with an eightfold rotational symmetry [7,8,9,10,11,12,13]. Accordingly, they are composed of a central scaffold (also called central framework or spoke complex), which is continuous with the cytoplasmic and the nucleoplasmic ring moieties and which is enclosing a central pore. Eight cytoplasmic filaments are connected to the cytoplasmic ring, whereas, in the nucleoplasm, eight filaments emanate from the nuclear ring and are brought together to a distal ring to form the nuclear basket.

**Figure 1:**
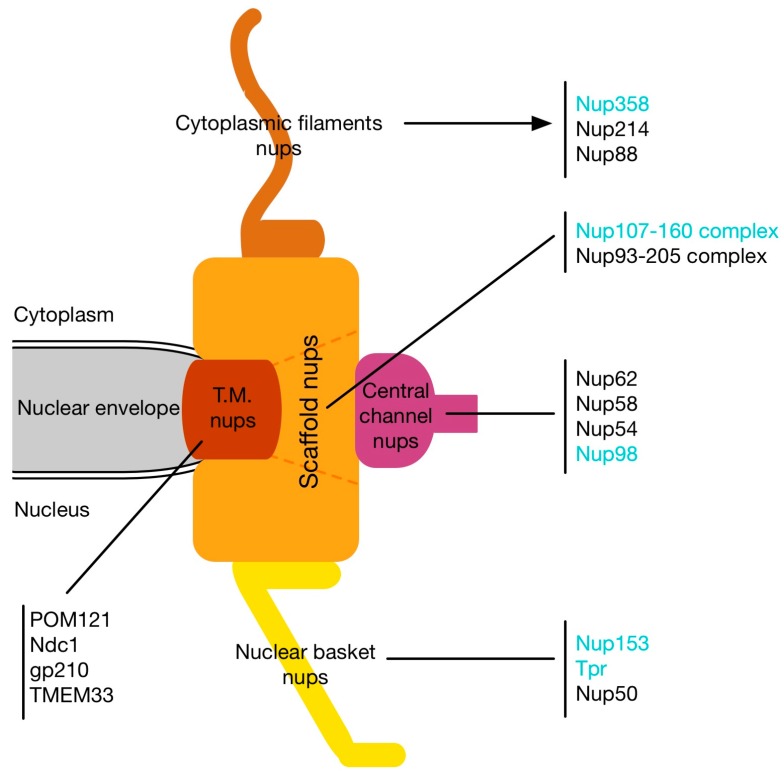
Schematic representation of nuclear pore complexes and the position of nucleoporins. The localization of the different nucleoporin subgroups is represented: Transmembrane nucleoporins (T.M. nups; **red**), scaffold nucleoporins (**orange**), central channel nucleoporins (**purple**), cytoplasmic filaments nucleoporins (**light brown**), and the nuclear basket nucleoporins (**yellow**). The nucleoporins highlighted in cyan refer to the nucleoporins implicated in the different mitotic processes discussed in this review.

Despite their enormous molecular weight, NPCs are consistently composed of only about 30 different proteins, the nucleoporins, in, for example, human cells [14,15], the budding yeast *Saccharomyces cerevisiae* [15], the fission yeast *Schizosaccharomyces pombe* [16], and the plant *Arabidopsis thaliana* [17,18]. The nucleoporins can be roughly categorized into three groups (Figure 1): the transmembrane, the structural, and the transport nucleoporins. In vertebrates, the transmembrane group comprises Ndc1, gp210, POM121, and TMEM33/POM33, which all have transmembrane domains that enable the link of NPCs to the NE [14,19,20,21,22]. Scaffold nucleoporins include the Nup107-160 complex, which is composed of nine components [23,24,25,26,27] and the Nup93/Nup205 complex, which contains five subunits [28,29,30]. Transport nucleoporins are found at the periphery of the NPCs and close to the central pore: The phenylalanine-glycine (FG) repeat containing Nup358 and Nup214 as well as Nup88 make the cytoplasmic filaments [31,32,33,34], whereas the nuclear basket is composed of the large coiled-coil nucleoporin Tpr (translocated promoter region) [35,36,37] and two FG nucleoporins Nup50 [38] and Nup153 [39,40,41,42,43]. Centrally located are the FG nucleoporins of the Nup62 complex [44,45] and Nup98 [46,47,48].

NPCs are gateways to the nucleus, which allow passive translocation of smaller molecules (<40 kDa) and mediate active, nuclear transport receptor (NTR)-dependent transport of larger molecules. NTRs are called karyopherins-β and they facilitate transport by weak interactions with the FG-repeat containing nucleoporins [49]. These karyopherins-β, also termed importins or exportins depending on their specific function, form cargo-receptor complexes by recognizing, respectively, the nuclear localization signal (NLS) and nuclear export signal (NES) of their cargo molecules [50,51]. Subsequently, the transport complexes interact with FG nucleoporins to be ferried across the NPC. Nucleocytoplasmic transport is controlled by the small Ras-related GTPase Ran, which is found in two different states: in a GTP-bound form (RanGTP) or GDP-bound (RanGDP) [52,53,54,55]. RanGTP is predominantly present in the nucleus and releases the import cargo from its receptor by an interaction with the importin, once the import complex has reached the nucleus. By contrast, RanGTP promotes binding of an export cargo to an exportin; the export complex passes the NPC in a similar fashion as import complexes, and is disassembled at the cytoplasmic face of the NPC due to GTP hydrolysis to generate RanGDP [50,51,52,56]. RanGDP is then reimported by its own import receptor, NTF2 (nuclear transport factor 2), and GTP is reloaded on Ran by the chromatin-bound guanine nucleotide exchange factor, RCC1 (regulator of chromatin condensation 1) [50,51,52,56].

Recent years have seen a growing appreciation for the role of NPCs and their constituents in a variety of cellular pathways other than nucleocytoplasmic transport, in both interphase and mitosis, such as the regulation of chromatin structure and gene expression [57,58,59,60,61], DNA repair [62,63], cell division, kinetochore assembly, and spindle assembly (for review see: [1,64,65,66]). Here, we will describe the link between NPCs, nucleoporins, and the spindle assembly checkpoint (SAC) in detail. We will discuss common characteristics and highlight species-specific differences in the interplay between these two cellular complexes.

## 2. Mitotic Functions of Nucleoporins and the Spindle Assembly Checkpoint

### 2.1. Nucleoporins at Kinetochores

In early stages of mitosis, after nuclear envelope breakdown (NEBD) and the disassembly of NPCs, several components of metazoan NPCs are re-localized to specific mitotic structures, such as the mitotic spindle, kinetochores, and centrosomes, to carry out active mitotic functions [64]. In this context, nucleoporins of the Nup107-160 complex as well as Nup358 were found at kinetochores to ensure the proper progression of the cell cycle. Kinetochores, which are large structures composed of around 90 different proteins, assemble on the centromere region of each chromosome, when chromosomes start to condense in prophase [67,68,69]. Spindle microtubules are reorganized from centrosomes after NEBD and NPC disassembly in prophase [70]. Kinetochores will then first interact with the lateral surface of spindle microtubules, termed a lattice [71]. To facilitate this interaction, kinetochores frequently generate microtubules [71]. Subsequently, the plus-ends of microtubules attach to kinetochores and the stable end-on attachments are essentially dependent on the KMN protein network (Knl1-Mis12 complex-Ndc80 complex), which displays microtubule-binding activity at kinetochores [68]. Moreover, microtubule-kinetochore attachment is regulated by reversible phosphorylation of kinetochore proteins, such as the KMN protein network, mainly via the Aurora B kinase and the phosphatase B56-PP2A to correct erroneous attachments [68]. The interaction between the kinetochores and the dynamically growing and shrinking microtubules has to be maintained constant for proper chromosome movement and segregation, which requires further factors that are capable of retaining the Ndc80 complex at the microtubule plus-ends, such as the spindle- and kinetochore-associated (SKA) complex in metazoans [68].

The Nup107-160 complex is the largest subunit of the NPC and formed of nine components, Nup160, Nup133, Nup107, Nup96, Nup85, Nup43, Nup37, Seh1, and Sec13, in conjunction with the putative transcription factor ELYS/Mel28 [23,25,72]. The Nup107-160 complex is critical for NPC assembly [24,26,72,73,74] and relocates to kinetochores after NPC disassembly, where it remains until anaphase [25,27,75]. While the partial depletion of Nup133 or ELYS by small interfering RNAs (siRNAs) in HeLa cells resulted in defective completion of cytokinesis without displacing the Nup107-160 complex from kinetochores [72], depletion of Seh1 by siRNA in mammalian cells led to a detachment of the entire complex and resulted in misaligned chromosomes and impaired chromosome segregation together with a delay in the progression from metaphase to anaphase [75,76]. Seh1 down-regulation further perturbed the centromeric localization of chromosome passenger complex (CPC) proteins, in particular the Aurora B kinase, which was suggested to be causative for the misalignment of the chromosomes in the respective cells [76]. Furthermore, the Nup107-160 complex appears to play a role in microtubule nucleation from unattached kinetochores, at least in HeLa cells. It interacts with at least two components of the γ-tubulin ring complex (γ-TuRC), GCP2 and GCP3 [77]. The γ-TuRC is an essential regulator of microtubule nucleation and is recruited to kinetochores during mitosis. Depletion of any member of the Nup107-160 complex led to a loss of the γ-TuRC from kinetochores, whereas depletion of GCP2 did not affect recruitment of the Nup107-160 to kinetochores [77]. Depletion of either the Nup107-160 complex or the γ-TuRC, however, prevented microtubule nucleation at kinetochores, indicating that Nup107-160 is required to recruit the γ-TuRC to kinetochores and for subsequent microtubule nucleation and spindle assembly. This occurs in a RanGTP-dependent manner [77]. Hence, the Nup107-160 complex has a dual role during mitosis: it regulates the spatial positioning of chromosomes by localizing the CPC to kinetochores and spindle assembly by recruiting γ-TuRC to kinetochores to allow microtubule nucleation.

The nucleoporin Nup358, also called RanBP2, is a large multidomain protein, which constitutes the cytoplasmic filaments of the NPCs and exhibits a SUMO E3 ligase activity [31,78]. In this context, Nup358 forms a complex with SUMO1-modified RanGAP1 and Ubc9, the E2 SUMO-conjugating enzyme [79,80,81,82]. When cells undergo mitosis, the Nup358-RanGAP1-Ubc9 complex adopts mitotic functions: recruited to kinetochores after initial microtubule attachment, it is required for the stability of kinetochore–microtubule attachments and, in turn, spindle assembly and sister chromatid segregation [83,84,85]. Necessary for positioning the Nup358-RanGAP1-Ubc9 complex at kinetochores are, on the one hand, the exportin CRM1 and RanGTP [84,85,86] as well as the presence of the Nup107-160 complex at kinetochores [75,76]. Upon NEBD, the level of RanGTP at kinetochores is elevated, which thus contributes to microtubule nucleation from kinetochores in early mitosis [77,87,88]. The high RanGTP concentration is maintained by its constitutive production by RCC1 [85] and by the displacement of RanGAP1 from kinetochores [89,90]. The delocalization of RanGAP1 is provoked by importin-β, which interacts with the *N*-terminal part of Nup358 and which persists in mitosis after NPC disassembly [89,90]. Importin-β therefore impedes the diffusion of RanGAP1 into the mitotic cytoplasm and in turn facilitates the recruitment of a transient RanGAP1–Nup358–CRM1–RanGTP complex to kinetochores upon microtubule attachment [89,90]. In contrast to importin-β, CRM1 and RanGTP positively regulate the recruitment of RanGAP1 to kinetochores, thereby promoting RanGTP hydrolysis, which in turn stops microtubule nucleation from kinetochores [89]. Thus, the mitotic localization of RanGAP1 and its binding to Nup358 is dependent on the balance between importin-β and CRM1 [89] and Nup358 is hence required for correct mitotic progression and faithful chromosome segregation in ensuring the localization of RanGAP1 during mitosis [89,90]. Depletion of Nup358 results in misaligned chromosomes, multipolar spindles, and the mis-localization of several kinetochore-associated proteins [83,84]. A study in mice further demonstrated the need for Nup358 for proper chromosome segregation [91]. This study revealed that Nup358 is important for the sumoylation and the subsequent recruitment of DNA topoisomerase IIα (TopoIIα) to centromeres. TopoIIα decatenates sister centromeres prior to anaphase onset, which is required for their separation after proper microtubule attachment [91,92]. Low amounts of Nup358 in mice led to failed recruitment of TopoIIα to centromeres and, as a consequence, chromosome separation defects such as DNA bridges during anaphase, which resulted in aneuploidy and tumor formation in mice, suggesting that Nup358 has tumor-suppressing activity [91]. In addition, Nup358 displays an essential function in sumoylation of the CPC component borealin in early mitosis. Nup358 was co-immunoprecipitated with borealin from a mitotic HeLa cell extract [93] and it is required for stimulating the sumoylation of borealin *in vitro*. Furthermore, sumoylation of borealin was nearly completely lost in HeLa cells depleted of Nup358, which could be restored by adding back a fragment of Nup358 that preserved its E3 SUMO ligase activity [93]. Sumoylation of borealin, however, is not required for regulating its localization as part of the CPC [93].

### 2.2. Nucleoporins, the SAC, and the APC/C

The examples of the Nup107-160 complex and Nup358 highlight the contribution of nucleoporins to proper mitotic progression, in particular to spindle assembly. However, although the Nup107-160 complex and Nup358 are localized to kinetochores during mitosis, these nucleoporins appear to not be involved in the regulation of the spindle assembly checkpoint (SAC, also named mitotic checkpoint), which occurs at kinetochores. Somewhat inconsistently, SAC regulation and the regulation of the anaphase-promoting complex/cyclosome (APC/C) are mediated by nucleoporins that are not recruited to kinetochores during mitosis, namely Tpr, Nup153, and Nup98. The SAC is the machinery that ensures faithful segregation of the genetic material into daughter cells by controlling the correct attachment of microtubules to kinetochores, via two major constituents, Mad1 and Mad2. The SAC is evolutionary highly conserved from yeast to higher eukaryotes and comprises, besides Mad1 and Mad2, another four components: Bub1, BubR1, Bub3, and Mps1 [94]. The target of the SAC is Cdc20, a cofactor invoking the E3 ubiquitin ligase activity of APC/C, which is required for metaphase–anaphase transition. The association of SAC proteins with Cdc20 at kinetochores during early mitosis prevents APC/C activation in generating the “wait anaphase” signal [94]. The SAC consists in the recruitment of a Mad1–Mad2 “core complex” to unattached kinetochores, which is pivotal for anaphase inhibition until all kinetochores are properly attached to microtubules [68,94]. This Mad1–Mad2 “core complex” is formed by binding Mad1 to Mad2, which provokes a conformational change of open-Mad2 (O-Mad2) to closed-Mad2 (C-Mad2) and acts as a receptor for further free O-Mad2 [94]. This transient interaction of C-Mad2–O-Mad2 also triggers the conformational change of O-Mad2 to C-Mad2 [94,95]. Subsequently, C-Mad2 forms a complex with BubR1 and Bub3 that binds to Cdc20 to inhibit APC/C [94,96]. Thus, the SAC hampers APC/C activation by Cdc20 to prevent untimely anaphase onset. For checkpoint turnoff and APC/C activation, Mad1 and Mad2 need to be released from kinetochores [94]. Once removed, the downstream targets of the activated APC/C, anaphase inhibitors securin and cyclin B, become ubiquitinated and degraded by the 26S proteasome. The degradation of securin frees its complex partner separase, a cysteine protease, which in turn cleaves the cohesin complex. Cohesin cleavage leads to separation of the sister chromatids and triggers the transition from metaphase to anaphase [97,98].

The SAC is therefore understood as critical to preserve genome integrity and its core components, Mad1 and Mad2, were interestingly found to reside at NPCs throughout interphase [99]. Both proteins are docked at the nuclear side of NPCs through interactions primarily with the nuclear basket elements Nup153 [100] and Tpr [101]. Tpr tethers both Mad1 and Mad2 at NPCs and this is conserved in most species [99,101,102,103,104,105,106], while Nup153 binds exclusively Mad1 [100] (for more details see Section 3.1.). Depleting Tpr from human cells resulted in the displacement of Mad1 and Mad2 from the NE during interphase and provoked mitotic defects, such as a more rapid exit from mitosis and lagging chromosomes [107,108]. Tpr is undetectable at spindle microtubules and kinetochores in human cells [107,108], whereas homologs of Tpr in *Drosophila* and *Aspergillus nidulans*, Mtor and Mlp1, respectively, were found in the proximity of spindle microtubules during mitosis [102,105,109]. Similar to Tpr, Nup153 is not associating with any mitotic substructure in human cells [100,110,111]. Overexpression of Nup153 in cultured human cells, however, lead to several mitotic abnormalities, including lagging chromosomes, cytokinesis failure, and override of the SAC. Nup153 interacts directly with Mad1 and its depletion reduces the pool of Mad1 at interphase NPCs [100]. How Tpr and Nup153 affect the SAC on a mechanistic level has remained largely elusive and deeper insight is only gradually arising (see below Section 3.2.; [107,108,112]).

Another cofactor of the APC/C is Cdh1 (Cdc20 homologue 1) [113,114]. Cdh1 activates APC/C (APC/C^Cdh1^) in late mitosis, which leads to the proteosomal degradation of securin and Cdc20 in vertebrates [115,116]. The activation of APC/C^Cdh1^ implicates the nucleoporin Nup98 in complex with its partner, the nuclear transport factor Rae1 [117]. An interaction of Nup98 and Rae1 with APC/C^Cdh1^ prevents unscheduled securin degradation, as combined Nup98 and Rae1 haploinsufficiency in mice lead to premature separation of sister chromatids and securin degradation already in prometaphase [117]. Moreover, this study has shown that the release of Nup98–Rae1 from APC/C^Cdh1^ occurs coincidentally with the release of BubR1 from APC/C^Cdc20^, which activates APC/C^Cdc20^ and APC/C^Cdh1^ to initiate metaphase–anaphase transition [117]. Thus, Nup98 and Rae1 are temporal regulators of APC/C^Cdh1^. Furthermore, Rae1 recognizes a small conserved motif called GLEBS (GLE2-binding-sequence) in Nup98 [118], and such a GLEBS motif is also found in the two SAC proteins Bub1 and BubR1, which both inhibit APC/C^Cdc20^ and are partners of Bub3, which displays homologies with Rae1 [119]. In addition to Nup98, Rae1 interacts with the GLEBS domain of Bub1 and along with Bub1 it localizes at kinetochores during prometaphase, further underscoring Rae1’s importance as a regulator of mitosis [119].

A further link between Nup98 and the APC/C came from a recent study on oncogenic Nup98 fusion proteins. The *NUP98* gene is a frequent target for chromosomal translocations, which fuse the *N*-terminal FG domain of Nup98 to the *C*-terminal domain of more than 30 different partners and are primarily causing acute myeloid leukemia (*AML*) [120,121]. In HEK293 human embryonic kidney cells Nup98 fusion proteins, unlike Nup98, were found to bind to APC/C^Cdc20^, thereby competing with BubR1, which shares a structural similarity with the *N*-terminal part of Nup98 [122]. Binding of Nup98 fusion proteins to APC/C^Cdc20^ interferes with SAC function. It alters APC/C^Cdc20^ activity and causes premature securin degradation, which leads to mitotic spindle defects and mis-segregation of chromosomes [122]. Whether or not this is related to AML remains to be seen, as patients typically have, despite the translocation, a normal karyotype at the time of disease diagnosis (see for example: [123,124,125,126]).

## 3. The SAC Regulators Mad1 and Mad2 during Interphase

### 3.1. Mad1 and Mad2 Localization at NPCs

In a *Xenopus laevis* fibroblast cell line, Mad1 and Mad2 were initially located at the NE during interphase [127], which by immunofluorescence experiments in HeLa cells was specified as localization at NPCs [99]. The recruitment of Mad1 and Mad2 to the NE occurs in two steps, as seen in *Drosophila* embryonic nuclei: when cells exit mitosis, Mad1 and Mad2 are first imported into the reforming nucleus before the association with the NE takes place [128]. The localization of Mad1 and Mad2 at NPCs during interphase appears to be evolutionarily conserved and has been observed in diverse species such as *Drosophila melanogaster* [128,129], the filamentous fungus *Aspergillus nidulans* [102], the yeasts *Saccharomyces cerevisiae* [104,106] and *Schizosacchoromyces pombe* [130], the nematode *Caenorhabditis elegans* [131], and the plant *Arabidopsis thaliana* [103]. However, the fate of NPCs in mitosis is different depending on these species. While eukaryotes such as humans and *Arabidopsis thaliana* undergo an open mitosis with NEBD coinciding with NPC disassembly, lower eukaryotes, such as the yeasts *Saccharomyces cerevisiae* and *Schizosacchoromyces pombe*, undergo a closed mitosis during which the nucleus and NPCs remain intact. In addition, other lower and higher eukaryotes are known to undergo a semi-open mitosis: in *Drosophila melanogaster* embryos, only NPCs are disassembled, so that the chromosomes remain enclosed by a NE. In *Aspergillus nidulans*, NPCs are partially disassembled during mitosis; only the FG nucleoporins are dispersed, which is thought to increase the permeability, whereas the remaining nucleoporins stay at NPCs [132,133]. *Drosophila* and *Aspergillus* therefore modify the permeability of the NE/NPCs without NEBD [134]. Similarly, *C. elegans* early embryos undergo such a semi-open mitosis [135].

While, as outlined above, Tpr [101] and Nup153 [100] were accordingly identified as the major binding partner of Mad1 at NPCs in human cells, the precise interaction partner of Mad2 is less clear. Some studies revealed that Tpr is also binding Mad2 [101], whereas this could not be confirmed in others [102,103]. Nevertheless, Tpr appears to be the prime binding partner for the SAC proteins at NPCs in most species, but not in all and not the sole. In *S. cerevisiae*, Mlp1p/Mlp2p, the yeast homologs of Tpr, were identified as Mad1p’s anchor at NPCs, whereas Mad2p’s partner is still unknown [106]. Besides the Mlp proteins, Nup53p, a scaffold nucleoporin, was identified as interaction partner of Mad1p at NPCs [104]. Mutational studies on the Ran GTPase pathway demonstrated that Gsp1p, the yeast ortholog of Ran, is implicated in the localization of Mad2p, but not Mad1p, at NPCs [136]. A reduction of the nuclear RanGTP pool triggered the release of Mad2p from NPCs, which coincided with the activation of the SAC [136]. In *Arabidopsis*, NUA, the Tpr homolog, binds Mad1 but not Mad2 [103]. Mad2 recruitment to NPCs occurs via Mad1, which interacts with NUA and Mad2 at the same time. The NPCs in plants, however, are less well understood as compared to their yeast and vertebrate counterparts [137,138] and future studies might therefore identify more nucleoporins that are required for targeting SAC proteins to NPCs in plants. Similarly, the parasite *Trypanosoma brucei*, a eukaryote that probably diverged early after the last eukaryotic common ancestor, is known to have NPCs and the nucleoporin TbNup92 displays analog functions to Mlp/Tpr proteins and is therefore also called TbMlp2 [139,140]. TbMlp2 is implicated in spindle association and chromosomal segregation during mitosis [139,140]. While the basic machinery of chromosome segregation is conserved in *Trypanosoma brucei*, no SAC proteins, except Mad2, have been identified thus far [141]. TbMad2 was found at the flagellar basal body of the parasite, but not in the nucleus or at kinetochores. It lacks a Cdc20-binding motif and does not co-purify with APC/C [141]. Thus, a functional SAC has so far not been revealed in *Trypanosoma brucei* [141].

In *Aspergillus*, Mlp1 binds Mad1, by which it tethers Mad2 to NPCs in interphase, and it is also necessary to maintain Mad1 in close proximity to kinetochores, but not for the targeting of Mad1 to the kinetochores [102]. Two other *Aspergillus* NPC constituents are necessary to recruit Mad1 to NPCs: Nup2, the ortholog of the vertebrate nuclear basket protein Nup50, and NupA [142]. Indeed, NupA was only recently identified as nucleoporin and it is required for the localization of Nup2 to NPCs in interphase. The sequence of NupA resembles none of the known nucleoporins, but it shows some functional similarities to human Nup153 and *S. cerevisiae* Nup1p [142]. NupA and Nup2 are both required for the nuclear import of Mad1 in the nuclei during the G1 phase of the cell cycle [142].

In *C. elegans*, it is Nup107 (Npp-5 in *C. elegans*), which appears as strictly required to localize Mad1 (MDF-1 in *C. elegans*) to the NE, although it is not required for SAC signaling [131]. In Npp-5-depleted embryos, a GFP-MDF-1 fusion protein as well as the endogenous MDF-1 failed to accumulate at the NE. In addition, a yeast two-hybrid assay revealed direct interaction between Npp-5 and MDF-1, in contrast to Npp-19, the ortholog of Nup53p [131]. Npp-5 has, moreover, a synthetic lethal interaction with MDF-1 and MDF- 2, the *C. elegans* Mad2, but the exact function of Npp-5 in SAC and cell cycle regulation has remained elusive.

Taken together, the interphase localization of Mad1 and Mad2 at the NE and NPCs is evolutionarily conserved from yeast to plants and higher eukaryotes. The anchoring of these SAC proteins at NPCs is species-specific (Table 1), although in the majority of species Tpr is the main anchoring site, at least for Mad1. Mad2 anchoring at NPCs is less well understood, but altogether the data suggest that the indispensable link between NPCs and the SAC machinery is critical for faithful cell division and chromosome segregation.

**Table 1:** Summary of known Mad1- and Mad2-interacting nucleoporins in distinct species.

Species	Localization of Mad1 and Mad2 at NPCs	Nucleoporins Partners
*Arabidopsis*	√	NUA (Tpr)
*Saccharomyces cerevisiae*	√	Mlp1p/Mlp2p (Tpr)
		Nup53p
*Schizosaccharomyces pombe*	√	No partners identified yet
*Aspergillus nidulans*	√	Mlp1 (Tpr)
*Drosophila melanogaster*	√	Mtor (Tpr)
*Caenorhabditis elegans*	√	Npp-5
Human	√	Tpr
		Nup153

### 3.2. Function of SAC Proteins at NPCs

**Figure 2:**
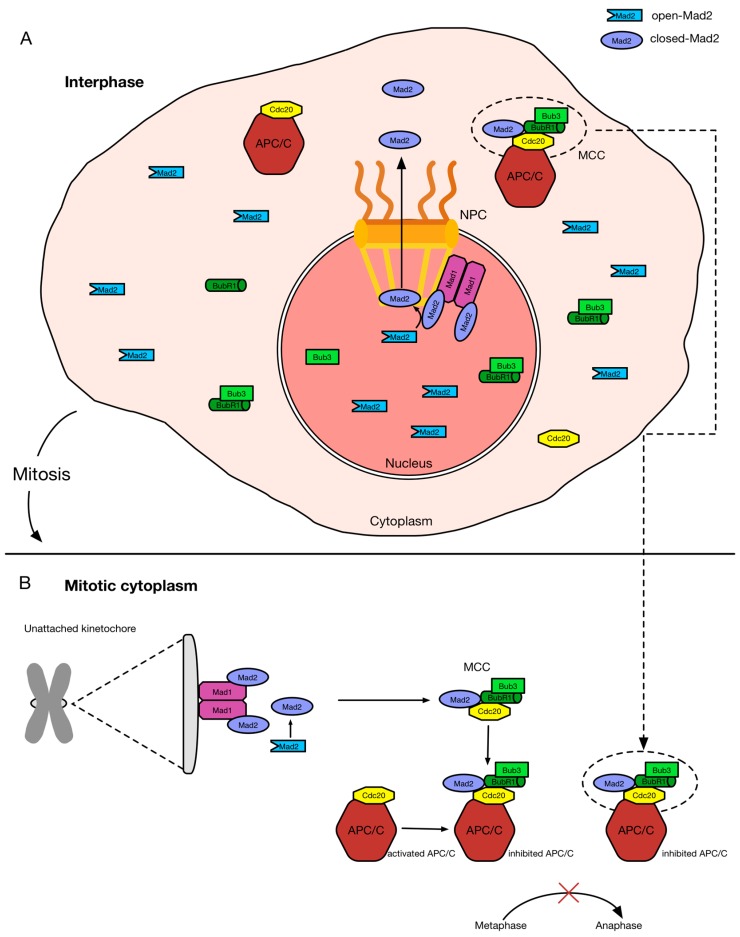
Schematic representation of MCC formation during interphase and mitosis. Mad1 and Mad2 form heterodimeric complexes in interphase and mitosis, which are localized to two distinct scaffolds: (**A**) the nuclear basket of NPCs; and (**B**) unattached kinetochores, respectively. The Mad1–Mad2 “core complex” provokes the conformational change of open-Mad2 (O-Mad2) to closed-Mad2 (C-Mad2). In this form, Mad2 binds to Cdc20 and with Bub3 and BubR1, it forms the MCC to inhibit the APC/C [143]. While unattached kinetochores catalyze the production of the MCC in mitosis, NPCs promote the production of the MCC in interphase [107]. In this way, Mad2 could be exported from the nucleus and translocated across the NPC after the induction of its conformational change to form the MCC in the cytoplasm [107]. These MCCs produced in interphase and in mitosis allow for the delay of anaphase onset and a more efficiency maintenance of the genome.

While the localization of Mad1 and Mad2 at NPCs is not questioned, their function here has remained largely elusive. Recent studies in human cells aimed at providing insights into the role of Mad1 and Mad2 at NPCs and demonstrated that this promotes the formation of a mitotic checkpoint complex (MCC) in interphase [107]. The MCC, which is formed by Mad2, BubR1, Bub3, and Cdc20 (Figure 2), assures the inhibition of APC/C function [143,144]. The formation of the MCC is promoted by unattached kinetochores, which thus serve as a scaffold in early mitosis to keep the APC/C inactive (Figure 2B), but it is also present in the cytoplasm of interphase cells (Figure 2A) [143]. The formation of the MCC in interphase is dependent on the Mad1–Mad2 complex and its Tpr-mediated association with NPCs, at least in human cells [107]. In this context, the NPC was demonstrated to act as a scaffold for MCC signaling and the depletion of Tpr or Mad1 or the expression of a Mad1 mutant lacking its nuclear pore-targeting domain in human cells displaced the Mad1–Mad2 complex from NPCs, which abolished the formation of the interphasic MCC [107]. The combined MCCs allow for the delay of anaphase onset and lead to a more sensitive and stronger SAC [107]. Thus, the association of the Mad1–Mad2 complex with Tpr during interphase is a prerequisite for proper SAC function during mitosis to assure proper kinetochores attachment of microtubules and correct chromosome segregation.

In contrast to human cells, *S. cerevisiae* undergoes closed mitosis and in this context Mad1 appears to have another function at the NPCs: Mad1p binds Nup53p to downregulate Kap121p-mediated nuclear transport in the presence of defective microtubule–kinetochore interactions [145]. Mechanistically, this is achieved by a cycling of Mad1p between kinetochores and NPCs. In fact, when kinetochores are detached from microtubules, Mad1p is translocated from Mlp1p/Mlp2p to kinetochores by the aid of the exportin Xpo1p (the ortholog of human CRM1). While a pool of Mad1 plays its role in the SAC, another pool of Mad1 returns from kinetochores to NPCs. At NPCs it binds Nup53p to inhibit Kap121p-mediated nuclear transport, which in turn controls the environment of the mitotic spindle [145,146]. It was suggested that Mad1 in metazoan cells may have a similar role. The localization of CRM1 at NPCs in interphase and at kinetochores in prophase may in this context be important for the translocation of Mad1 from NPCs to unattached kinetochores in metazoan cells [146]. This hypothesis, however, requires further investigations for verification.

## 4. Conclusions

Beyond the role in the nucleocytoplasmic transport during interphase, some nucleoporins are of outmost importance at distinct steps of mitosis: for example, the Nup107-160 complex and Nup358 for proper spindle assembly, Tpr and Nup153 for the spindle assembly checkpoint, or Nup98 and its partner Rae1 for the APC/C. These nucleoporins are therefore contributing to successful cell division in avoiding spindle defects, the presence of unattached kinetochores during chromosome alignment, and/or untimed anaphase onset, and thus are ensuring proper segregation of sister chromatids and genome integrity. Moreover, the link between NPCs and SAC proteins is conserved across species, despite the fact that the exact anchoring of SAC proteins at NPCs is species-specific. Mad1 and Mad2 localization at NPCs in interphase ensures the formation of a MCC during interphase, which, in combination with the mitotic MCC, is required for the delay of anaphase onset. Whether or not Mad1 and Mad2 at NPCs fulfill other critical functions remains to be investigated.
